# Sodium-Based Cylindrical Plasmonic Waveguides in the Near-Infrared

**DOI:** 10.3390/nano12121950

**Published:** 2022-06-07

**Authors:** Da Teng, Yuanming Tian, Xuemei Hu, Ziyi Guan, Wencang Gao, Pengyuan Li, Hongli Fang, Jianjun Yan, Zhiwen Wang, Kai Wang

**Affiliations:** 1 College of Physics and Electronic Engineering, Zhengzhou Normal University, Zhengzhou 450044, China; tianyuanming_8426@163.com (Y.T.); hu19139279202@163.com (X.H.); guanziyi0505@163.com (Z.G.); gwc315760@163.com (W.G.); m13213001330@163.com (P.L.); fhl_liquid@163.com (H.F.); yjj_llxl@163.com (J.Y.); zwwang@zznu.edu.cn (Z.W.); 2 Key Laboratory of Infrared Imaging Materials and Detectors, Shanghai Institute of Technical Physics, Chinese Academy of Sciences, Shanghai 200083, China

**Keywords:** plasmonic waveguide, field confinement, near-infrared, quality factor

## Abstract

Subwavelength optical field confinement and low-loss propagation are of great significance for compact photonic integration. However, the field confinement capability of plasmonic devices is always accompanied by the inherent Ohmic loss. Although recent studies have shown that sodium (Na) exhibits lower loss than noble metals in the near-infrared band, the field confinement ability has not been adequately assessed. Meanwhile, the high chemical reactivity of Na should be regulated for practical application. Two dielectric-coated Na nanowires, consisting of cylindrical Na nanowires with one or two dielectric layers as claddings, are proposed and investigated in this paper. Based on finite element calculations, we thoroughly study the modal fields and low-loss propagation properties of dielectric-coated Na nanowires. The results demonstrate that Na exhibits lower loss and stronger field confinement than the typical plasmonic material silver. These findings indicate the performance of plasmonic devices can be considerably improved by employing the metal Na compared with devices using noble metals, which may promote the applications in subwavelength photonic devices.

## 1. Introduction

Surface plasmons (SPs) [1] have been considered as prospective candidates for the integration and miniaturization of photonic components and circuits, owing to their remarkable capabilities of squeezing light into regions far smaller than the diffraction limit [2,3]. Various noble metal-based designs have been suggested and investigated to support well-confined SP modes, including nanowires [4,5,6,7,8], slot waveguide [9,10,11], dielectric-loaded waveguide [12], long-range waveguide [13], and wedge waveguide [14], to mention a few. Due to the presence of metal (usually gold and silver), SP waveguides inevitably suffer from high propagation loss, and there exist trade-offs between losses and confinements for plasmonic waveguides stated above. These structures have either poor field confinement or high loss, hindering their applications where both subwavelength confinement and long-range propagation are needed.

To alleviate this conflict, hybrid plasmonic waveguides (HPWs) [15,16,17,18,19,20,21,22,23,24,25,26,27,28] have been proposed and demonstrated, which combine the benefits of both low-loss properties of dielectric fibers and strong field confinement of plasmonic waveguides. However, for practical applications, the loss of these structures is still unacceptably high. Thus, searching for better plasmonic materials [29,30,31] has become a hot topic. In recent years, researchers have suggested some available materials for supporting SPs, such as nitrides [31], transparent conducting oxide [32], graphene [33,34,35,36,37,38,39,40,41], transition metal dichalcogenides [42,43,44], bulk Dirac semimetals [45,46,47], borophene [48,49,50], etc. Although some of these materials support SP modes with strong field confinement and even tunable characteristics, the loss is still unsatisfactory, making them less suitable for real-life applications.

Quite recently, researchers reported [51] a plasmonic nanolaser based on alkali metal Na, which has a lasing threshold lower than previously reported at near-infrared wavelengths. In this work, researchers developed a thermo-assisted spin-coating process for fabricating a high-quality Na film and experimentally measured the dielectric function of Na in the wavelength range 400–1500 nm using a spectroscopic ellipsometer. Notably, the optical damping rate of Na is only half of that of silver. Hence, Na seems to be an ideal plasmonic material with much lower loss than gold and silver from ultraviolet to the near-infrared band. Then, research on propagating and localized Na plasmons was carried out. Tao et al. [52,53] proposed and studied directional couplers based on Na plasmonic waveguide. Abdelsalam et al. [54] investigated localized electromagnetic modes of Na nanoparticles. Recently, SPs were excited at the interface of sodium and graphene [55].

For plasmonic waveguides, both low-loss propagation and strong optical field confinement are of great importance. To our knowledge, although reports have shown that Na exhibits lower loss than gold and silver in the near-infrared band, the field confinement ability has not been properly assessed. Meanwhile, the high chemical reactivity of Na [51] should be controlled for practical application. Here, we propose and investigate two dielectric-coated Na nanowires, consisting of a cylindrical Na nanowire and one or two dielectric layers as claddings. We first compare the permittivity and quality factor of metal Na and Ag, then systematically study the modal field confinement ability and low-loss propagation properties. Meanwhile, we compare the subwavelength propagation properties of dielectric-coated Na and Ag nanowires. The findings reveal that Na nanowires exhibit not only lower loss, but also stronger field confinement than the typical plasmonic material silver, which makes it quite suitable to serve as building blocks for subwavelength photonic devices.

## 2. Theory

To quantitatively analyze the loss of Na- and Ag-based plasmonic waveguides, we need to obtain the dielectric permittivity. The permittivity of Ag (*ε*_Ag_) is adopted from Yang’s experimental data [56], while for Na, we use a Drude–Lorentz model to calculate the dielectric permittivity (*ε*_Na_), which is expressed as [51]:

(1) $$\varepsilon_{\mathrm{Na}}\left(\omega \right)=\varepsilon_{1}+i\varepsilon_{2}\;=\varepsilon_{b}-\frac{\omega_{p}^{2}}{\omega^{2}+i\omega \gamma_{p}}+\frac{f_{1}\omega_{1}^{2}}{\omega_{1}^{2}-\omega^{2}-i\omega \gamma_{1}}$$

where *ε*_b_ = 0.5, *ω*_p_ = 5.414 eV, *γ*_p_ = 0.01 eV, *f*_1_ = 0.28, *ω*_1_ = 2.945 eV, and *γ*_1_ = 2.706 eV.

**Figure 1:**
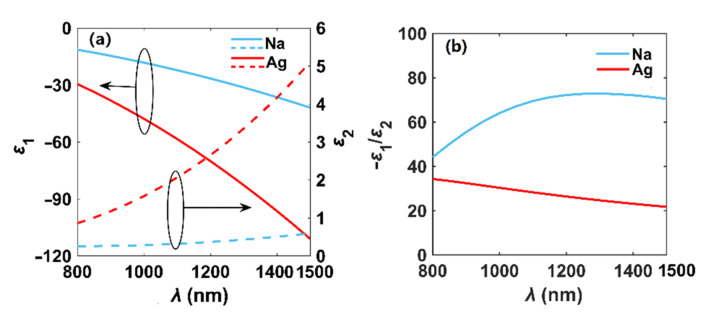
Permittivity (*ε* = *ε*_1_ + i*ε*_2_) (**a**) and quality factor (−*ε*_1_/*ε*_2_) (**b**) of Na and Ag. The solid and dashed lines of (**a**) stand for the real and imaginary parts of the permittivity, respectively.

The permittivity and quality factor (−*ε*_1_/*ε*_2_) of metal Na and Ag are compared in Figure 1 across the wavelength range of 800 to 1500 nm. As seen in Figure 1a, the absolute value of Re(*ε*_Ag_) is larger than that of Na, while Im(*ε*_Na_) (related to loss) is smaller than that of Ag, and the discrepancy grows as the wavelength increases. Thus, we conclude that the loss of Na is lower than that of Ag, especially at longer wavelengths. In Figure 1b, we see the quality factor of Na firstly increases with the increase in wavelength. When the wavelength exceeds 1200 nm, the quality factor of Na approaches its maximum value and then has a slight downward trend, while the quality factor of Ag monotonically decreases when *λ* ranges from 800 to 1500 nm.

Considering the fundamental plasmon mode (TM_0_) propagating in the *z*-direction with a complex propagation constant *β* = *n*_eff_*k*_0_, *k*_0_ = 2π/*λ*, with *λ* being the wavelength in vacuum, and *n*_eff_ = *n*_eff_r_ + i*n*_eff_i_ is the complex effective mode index. Propagation length is defined by *L*_P_ = 1/*k*_0_*n*_eff_i_ and normalized mode area is defined by $A_{N}=A_{\mathrm{eff}}/A_{0}=\iint W(r)d^{2}r/\max\{W(r)\}/(\lambda^{2}/4)$, where *W*(r) is the energy density of the plasmon mode [15]. Figure of merit (FoM) [57] is given by *L*_P_/(*A*_eff_/π)^1/2^. The results are obtained by use of the wave optics module of COMSOL Multiphysics, which is widely used for simulating plasmonic devices [58,59,60,61]. The eigenvalue solver is used to find modes of the waveguide. The calculation domain has a radius of 5*λ*, and a perfectly matched layer (PML) is applied at the outer boundary to keep away from the influence of reflection. A convergence analysis is also conducted to ensure that the numerical boundaries and meshing sizes do not interfere with the solutions.

## 3. Results and Discussion

### 3.1. Modal Properties in Dielectric-Coated Metallic Nanowires (DCMNW)

**Figure 2:**
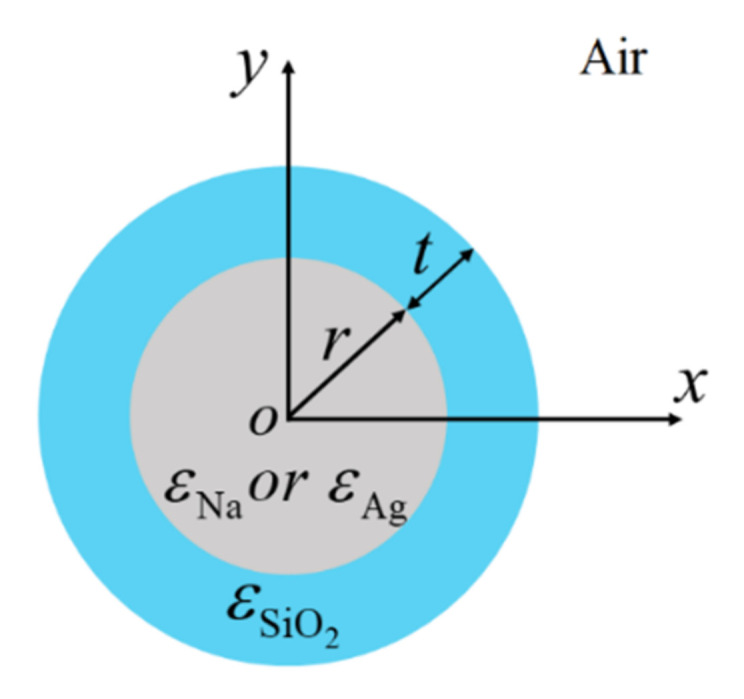
The cross-section of dielectric-coated Ag or Na nanowire.

**Figure 3:**
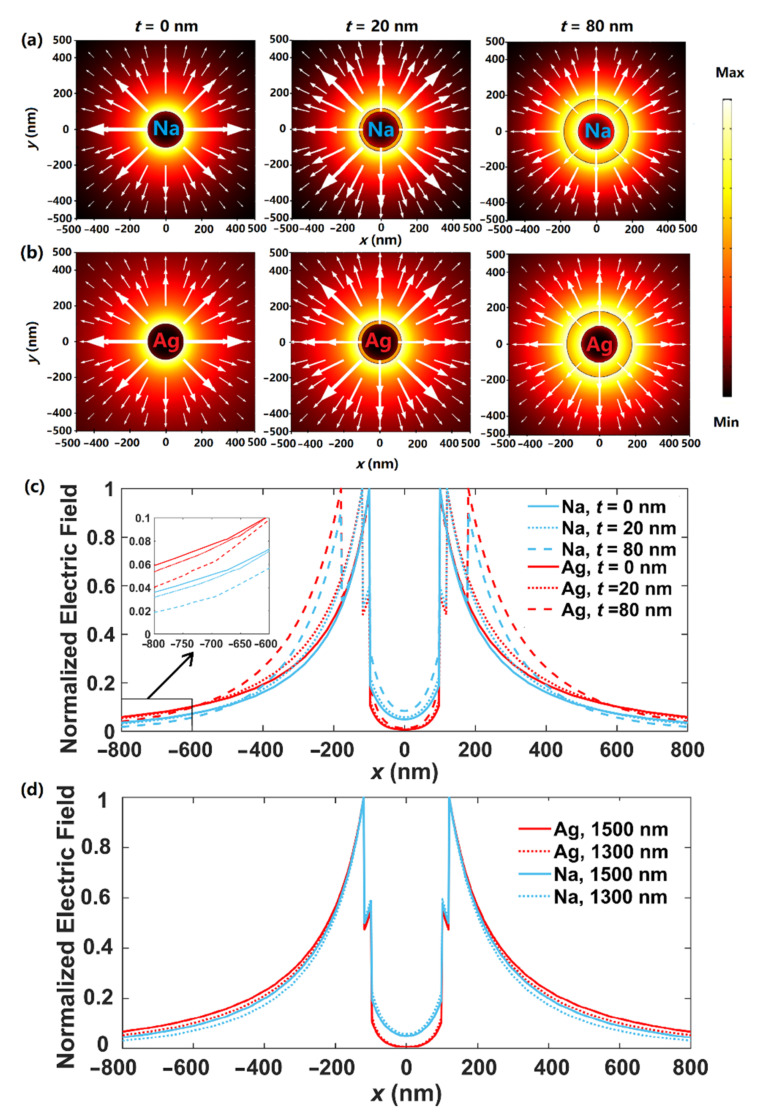
Field distributions of |**E**| of the fundamental mode at *λ* = 1300 nm for dielectric-coated (**a**) Na and (**b**) Ag nanowires at *t* = 0, 20, 80 nm. White arrows indicate radially polarized electric fields. (**c**) Field distributions along *x*-direction. (**d**) Comparison of field distributions along *x*-direction for *λ* = 1300 and 1500 nm with *t* = 20 nm.

**Figure 4:**
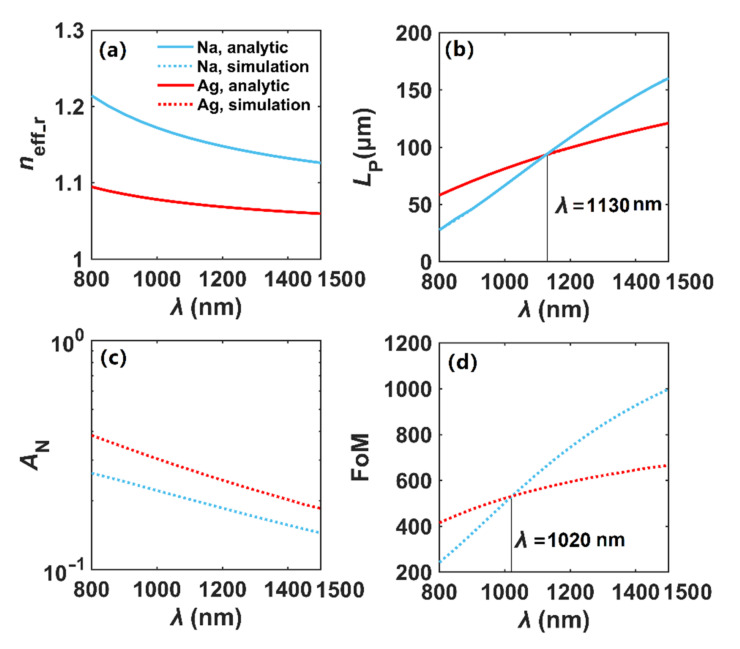
Modal properties of single Na and Ag nanowires in vacuum. (**a**) *n*_eff_r_, (**b**) *L*_P_, (**c**) *A*_N_, (**d**) FoM. Analytical results of *n*_eff_ are obtained from Equation (2) of Ref. [4].

**Figure 5:**
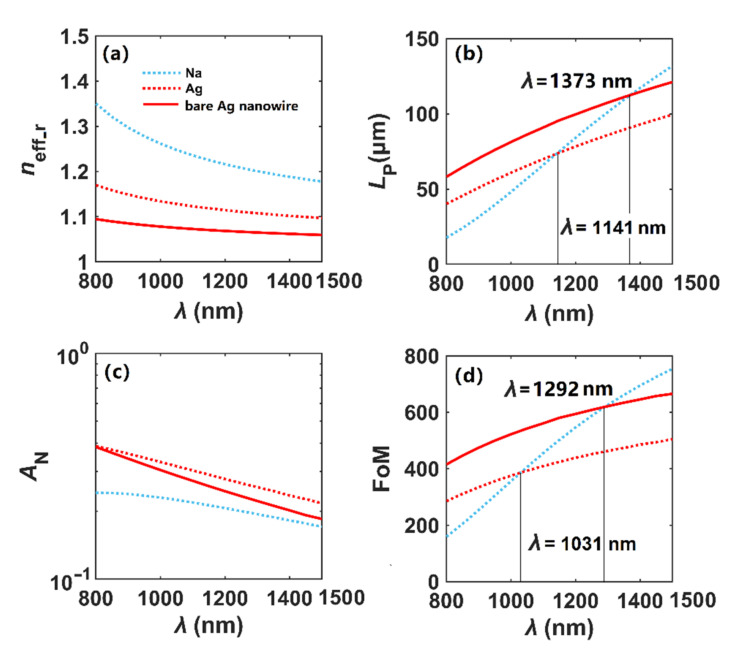
Modal properties of SiO_2_-coated Na and Ag nanowires. (**a**) *n*_eff_r_, (**b**) *L*_P_, (**c**) *A*_N_, (**d**) FoM.

**Figure 6:**
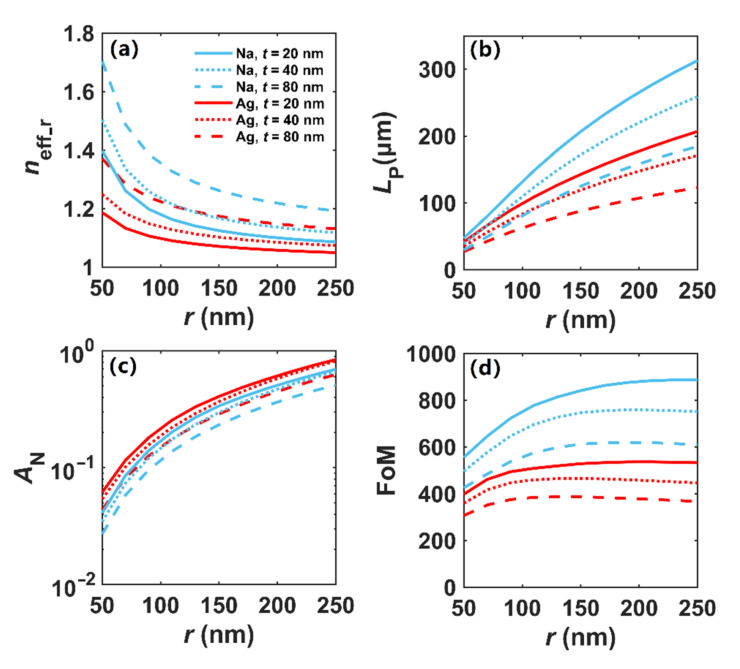
Modal properties of SiO_2_-coated Na and Ag nanowires with different radii. (**a**) *n*_eff_r_, (**b**) *L*_P_, (**c**) *A*_N_, (**d**) FoM.

Figure 2 depicts the schematic of DCMNWs, where *r* is the radius of the nanowire, and *t* is the thickness of the dielectric layer with permittivity of *ε*_SiO2_ = 2.25 [62]. The whole structure is located in vacuum with *ε*_0_ = 1. We first investigate the modal field distributions in DCMNWs by setting *λ* = 1300 nm, *r* = 100 nm, and *t* = 0, 20, 80 nm. The special case of *t* = 0 nm corresponds to a single metallic nanowire in vacuum. The 2D modal field distributions of dielectric-coated Na and Ag nanowires are shown in Figure 3a,b, respectively. Clearly, the fundamental plasmon mode (TM_0_ with field components of *E*_r_, *H*_φ_, *E*_z_) has a radially polarized electric field, which exponentially decays away from the metal surface. To obtain an intuitive observation, we plot the fields along the *x*-direction in Figure 3c. The field penetrates through the dielectric-coated Na nanowire, while the fields inside dielectric-coated Ag nanowires have zero value points. Furthermore, compared with that of Na, the field confinement is even weaker for the dielectric-coated Ag nanowire. For instance, the full width at half maximums (FWHMs) of the field plots corresponding to *t* = 0, 20, and 80 nm are 378, 432, and 570 nm for Ag, while 362, 416, and 509 nm for Na. More importantly, by increasing *t*, the field diffusion could be suppressed; that is, the field values (|x| > 600 nm) decrease as *t* increases, as shown in the inset of Figure 3c. Figure 3d compares the field distributions along the *x*-direction for dielectric-coated Na and Ag nanowires with *λ* = 1300 and 1500 nm, *t* = 20 nm, and *r* = 100 nm. As the wavelength increases, FWHMs of the field plots slightly enlarge. For *λ* = 1500 (1300) nm, FWHMs are 444 (432) nm for Ag and 428 (416) nm for Na. Thus, we can reasonably deduce that (i) the field confinement ability of dielectric-coated Na nanowires is stronger than that of dielectric-coated Ag nanowires (see Figure 4c, Figure 5c and Figure 6c); (ii) increasing the cladding thickness *t* results in a more restricted modal field (see Figure 6c).

In Figure 4, we evaluate the transmission characteristics of Na and Ag nanowires and perform the benchmark work by comparing the analytical results of *n*_eff_ [4] and the simulated ones, where *r* = 100 nm and *t* = 0 nm. As shown in Figure 4a, we can see the *n*_eff_r_ of the plasmon modes in Na and Ag nanowires decrease with increasing wavelength, and the solid lines and dot lines are highly overlapped, indicating that the simulated and analytical results are in good agreement. The propagation lengths increase for both Na and Ag nanowires, as shown in Figure 4b. However, when *λ* exceeds 1130 nm, the *L*_P_ of the mode in the Na nanowire is larger than that of the Ag nanowire. Meanwhile, as shown in Figure 4c, the modal field area of the fundamental plasmon mode in the Na nanowire is always smaller than Ag throughout the whole wavelength range, implying that the Na nanowire has stronger field confinement ability than the Ag nanowire. Finally, in Figure 4d, we can see that the FoM increases, and when *λ* exceeds 1020 nm, the FoM of Na-based plasmon mode is much larger. Therefore, the plasmonic effect of Na is better than Ag at longer wavelengths, which is consistent with the results described in Figure 1.

In Figure 5, we study the transmission characteristics of Na and Ag nanowires (*r* = 100 nm) coated with a silica layer of *t* = 20 nm at different wavelengths. A bare Ag nanowire is also investigated for comparison. As shown in Figure 5a, the *n*_eff_r_ of the plasmon modes in Na and Ag nanowires decreases with the increase in *λ*, while *L*_P_ increases with the increase in *λ* shown in Figure 5b. When *λ* exceeds 1141 nm, the *L*_P_ of the mode in the silica-coated Na nanowire is larger than that of the silica-coated Ag nanowire. When *λ* exceeds 1373 nm, the *L*_P_ of the mode in the silica-coated Na nanowire is even larger than that of a bare Ag nanowire. As in Figure 4c, it can be seen from Figure 5c that the modal field area of the fundamental plasmon mode in the silica-coated Na nanowire is smaller than both silica-coated and bare Ag nanowires throughout the entire wavelength range, indicating that the dielectric-coated Na nanowire has stronger field confinement ability than dielectric-coated and bare Ag nanowires. Finally, we can see from Figure 5d that the FoM increases, and when *λ* exceeds 1292 nm, the FoM of the Na-based plasmon mode is much larger. As seen in Figure 5b,c, the silica-coated Na nanowire outperforms its silver counterpart in both field confinement and propagation loss when *λ* > 1141 nm, and the silica-coated Na nanowire also outperforms the bare silver nanowire when *λ* > 1373 nm. As a result, *λ* is set to be 1500 nm in the following.

Then, with *r* varying from 50 to 250 nm, we investigate the modal transmission characteristics of Na and Ag nanowires coated with various thicknesses of silica, where *λ* = 1500 nm and *t* = 20, 40, and 80 nm. In Figure 6a, we observe that the *n*_eff_r_ of the plasmon modes in dielectric-coated Na and Ag nanowires decrease with the increase in nanowire radius, and *n*_eff_r_ of Na plasmon modes are larger than those of Ag under the same thickness of silica. When *r* > 200 nm, the effective mode index *n*_eff_r_ tends to be stable and decreases slowly. As shown in Figure 6b, the modal propagation lengths in both silica-coated Na and Ag nanowires increase with the increase in *r*, and Na nanowires have an advantage over Ag nanowires in the propagation distance with the same value of *t*. For instance, the *L*_P_ of silica-coated Na and Ag is about 313 μm (i.e., 208.6*λ*) and 207 μm (i.e., 138*λ*) when *r* = 250 nm and *t* = 20 nm, respectively. Meanwhile, as seen in Figure 6c, the modal field area of silica-coated Na nanowires is smaller than that of Ag when the thicknesses of SiO_2_ are set to be the same. By increasing the cladding thickness *t*, a more confined modal field could be achieved. Apparently, the trade-off between loss and field confinement still exists. When enlarging *r*, the field confinement weakens, while the propagation length increases. As shown in Figure 6d, the FoM of silica-coated Na could reach as high as 886, while that of silica-coated Ag is only 533 when *r* = 250 nm and *t* = 20 nm, and the FoM tends to be stable when *r* exceeds 150 nm.

### 3.2. Modal Properties in Double Dielectric-Coated Metallic Nanowires (DDCMNWs)

**Figure 7:**
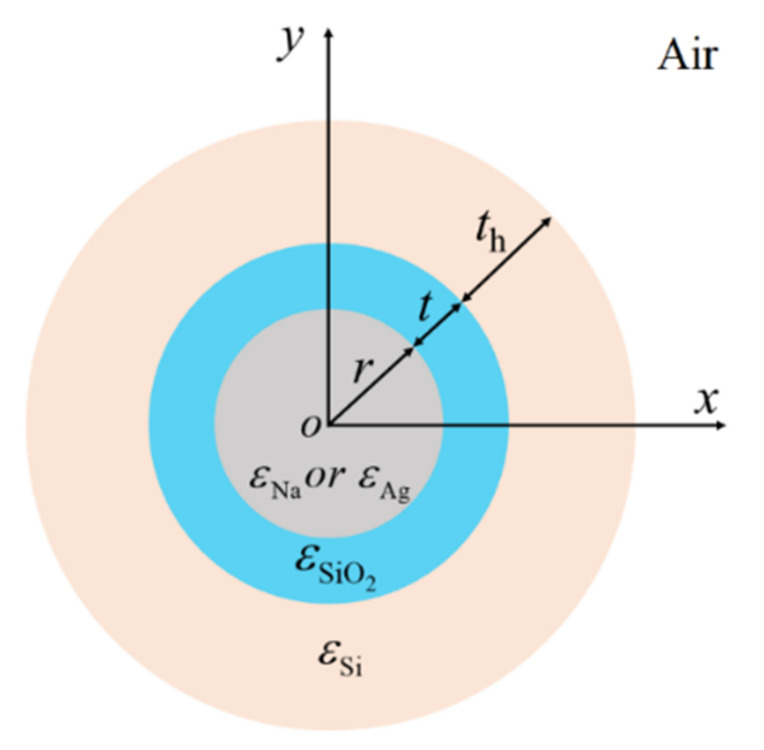
The cross-section of DDCMNWs based on Ag or Na nanowires.

**Figure 8:**
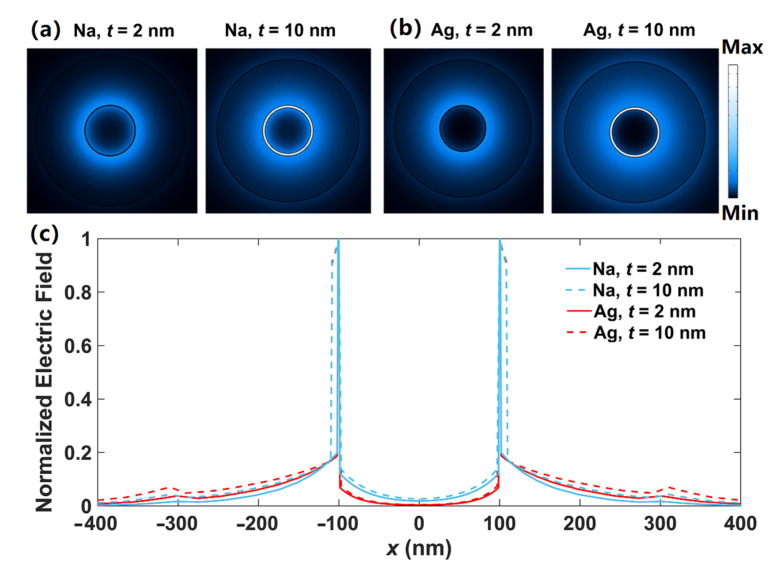
Field distributions of **|E|** of the fundamental mode in DDCMNWs at *λ* = 1500 nm for (**a**) Na and (**b**) Ag. (**c**) Field distributions along *x*-direction.

By adding another high-index dielectric layer to the DCMNWs, one can easily form the double dielectric-coated metallic nanowires (DDCMNWs), as shown in Figure 7. *t*_h_ is the thickness of the high-index dielectric layer with permittivity of *ε*_Si_ = 12.25 [63]. The modal field distributions in DDCMNWs are investigated by setting *λ* = 1500 nm, *r* = 100 nm, *t* = 2, 10 nm, and *t_h_* = 200 nm. The 2D modal field distributions of DDCMNWs based on Na and Ag are depicted in Figure 8a,b, respectively. Clearly, the optical energy is mainly concentrated inside the gap region between the metal nanowire and outside the high-index dielectric layer. As shown in Figure 8c, the field distributions inside the nanowires are similar to those shown in Figure 3c; that is, the fields penetrate through the Na nanowires, while the fields inside Ag nanowires have zero value points. Moreover, at the outside boundaries of the high-index materials, the field values of dielectric-coated Ag nanowires are larger than those of Na nanowires, which indicates the field confinement of the Ag-based DDCMNW is weaker compared with that of the Na-based DDCMNW.

**Figure 9:**
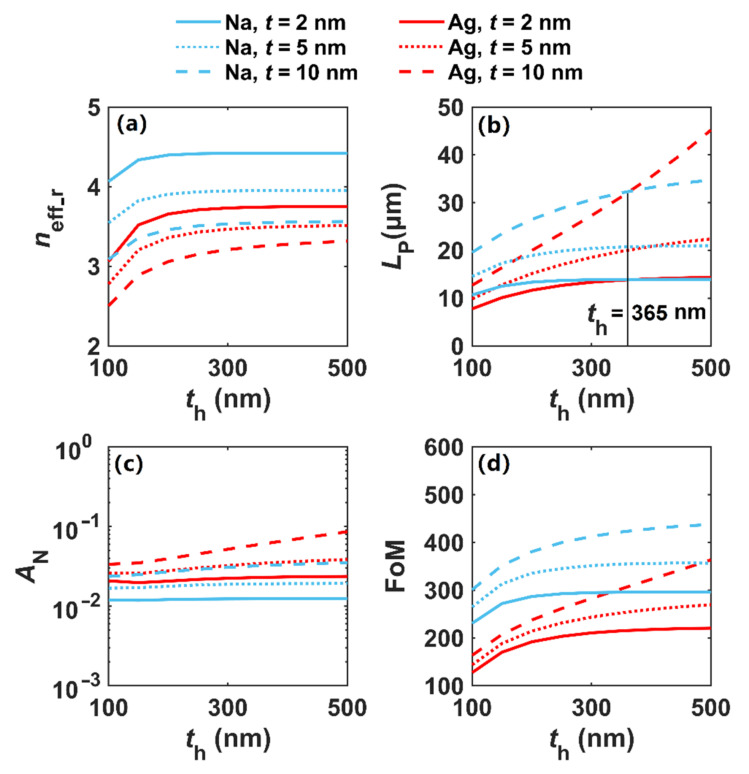
Modal properties of DDCMNWs based on Na and Ag with different cladding thickness *t*_h_ at *λ* = 1500 nm. (**a**) *n*_eff_r_, (**b**) *L*_P_, (**c**) *A*_N_, (**d**) FoM. Thicknesses of SiO_2_ are set to be *t* = 2, 5, 10 nm and *r* = 100 nm.

In Figure 9, we study the modal properties of DDCMNWs based on Na and Ag nanowires with different cladding thicknesses *t*_h_. As shown in Figure 9a,b, the *n*_eff_r_ of the plasmon modes in Na and Ag DDCMNWs firstly increases with the increment of cladding thickness *t*_h_ and then tends to be invariable when *t*_h_ > 150 nm, while the propagation length increases with the increment of *t*_h_. For a smaller gap distance, i.e., *t =* 2 and 5 nm, *L*_P_ tends to be stable when *t*_h_ > 300 nm. It is worth to be noted that when for *t*_h_ > 365 nm and *t* = 10 nm, the loss of the Ag-based DDCMNW becomes smaller than that of Na, whereas, the plasmon modal field area of the Na-based DDCMNW is smaller than those of Ag throughout the whole *t*_h_ range shown in Figure 9c. This indicates that the field confinement of the Na-based DDCMNW is superior to that of the Ag-based DDCMNW. Meanwhile, the smaller the gap distance, the stronger the field confinement. For *t*_h_ = 300 nm and *t* = 2, 10 nm, *A*_N_ is 0.0123 and 0.0307 for the Na-based DDCMNW, respectively. Finally, in Figure 9d, we can see that the FoM increases for both Na- and Ag-based DDCMNWs. The Na-based DDCMNW has a larger FoM than Ag for the same *t*. Therefore, the results show that the plasmonic effect of Na is better than that of Ag; that is, Na-based plasmons exhibit not only lower loss, but also stronger field confinement than the typical plasmonic material Ag when *t*_h_ < 365 nm.

Although SiO_2_-Si double dielectric-coated metal nanowires (DDCMNWs) exhibit relatively strong dissipation and significantly smaller propagation distances (Figure 9b) than SiO_2_ coated metal nanowires (DCMNWs) (Figure 6b), their major advantage is the possibility of stronger subwavelength confinement of the guided modes (smaller *A*_N_, see Figure 6c and Figure 9c), which is one of the major requirements for interconnects in highly integrated optical circuits and subwavelength optical devices.

Finally, we need to explain here that we only consider the comparison between Na and Ag for reasons listed below. Different metals have been employed to excite SPs in different frequency bands in the literature. For instance, copper is used in the terahertz band [64]. Aluminum (Al) possesses material properties that enable strong plasmon resonances spanning much of the visible region and into the ultraviolet [65], while silver and gold are often used in the visible region and near infrared [29]. In terms of SP waveguiding, silver provides a larger FoM than gold (see Figures 18 and 19 of Ref. [30]). As a result, we skip the comparison between Na and Au and conclude that Na-based plasmon modes outperform Ag- and Au-based plasmon modes in terms of subwavelength waveguiding.

## 4. Conclusions

We propose and investigate (double) dielectric-coated Na nanowires by using finite element calculations. Increasing the cladding thickness *t* of the dielectric-coated Na nanowires results in a more confined modal field. The silica-coated Na nanowire outperforms its silver counterpart in terms of field confinement and propagation loss when *λ* > 1141 nm and also outperforms the bare silver nanowire when *λ* > 1373 nm. The *L*_P_ and FoM of the silica-coated Na nanowire are about 208.6*λ* and 886 when *r* = 250 nm and *t* = 20 nm. For double dielectric-coated Na nanowires, the loss and normalized modal field area are both smaller compared with those of Ag-based nanowire waveguides when *t*_h_ < 365 nm. The obtained results suggest Na-based plasmons exhibit not only lower loss, but also stronger field confinement than the typical plasmonic material Ag at longer wavelengths, which implies the subwavelength waveguiding performance of plasmonic devices can be greatly improved by using Na. These results may have potential applications in the fields of subwavelength photonic devices, such as nanolasers, resonators, sensors, and other waveguide-integrated devices.

## Data Availability

The data presented in this study are available on request from the corresponding author.
